# Novel Sampling Method for Assessing Human-Pathogen Interactions in the Natural Environment Using Boot Socks and Citizen Scientists, with Application to Campylobacter Seasonality

**DOI:** 10.1128/AEM.00162-17

**Published:** 2017-06-30

**Authors:** Natalia R. Jones, Caroline Millman, Mike van der Es, Miroslava Hukelova, Ken J. Forbes, Catherine Glover, Sam Haldenby, Paul R. Hunter, Kathryn Jackson, Sarah J. O'Brien, Dan Rigby, Norval J. C. Strachan, Nicola Williams, Iain R. Lake, for the ENIGMA Consortium

**Affiliations:** a School of Environmental Sciences, University of East Anglia, Norwich, United Kingdom; b Department of Economics, School of Social Sciences, University of Manchester, Manchester, United Kingdom; c Norwich Medical School, University of East Anglia, Norwich, United Kingdom; d School of Medicine, Medical Sciences & Nutrition, University of Aberdeen, Aberdeen, United Kingdom; e Institute of Infection and Global Health, University of Liverpool, Liverpool, United Kingdom; f Centre for Genomic Research, University of Liverpool, Liverpool, United Kingdom; g Institute of Psychology, Health & Society, University of Liverpool, Liverpool, United Kingdom; h School of Natural and Computing Sciences, University of Aberdeen, Aberdeen, United Kingdom; University of Michigan—Ann Arbor

**Keywords:** boot socks, Campylobacter, citizen science, environmental sampling

## Abstract

This paper introduces a novel method for sampling pathogens in natural environments. It uses fabric boot socks worn over walkers' shoes to allow the collection of composite samples over large areas. Wide-area sampling is better suited to studies focusing on human exposure to pathogens (e.g., recreational walking). This sampling method is implemented using a citizen science approach: groups of three walkers wearing boot socks undertook one of six routes, 40 times over 16 months in the North West (NW) and East Anglian (EA) regions of England. To validate this methodology, we report the successful implementation of this citizen science approach, the observation that Campylobacter bacteria were detected on 47% of boot socks, and the observation that multiple boot socks from individual walks produced consistent results. The findings indicate higher Campylobacter levels in the livestock-dominated NW than in EA (55.8% versus 38.6%). Seasonal differences in the presence of Campylobacter bacteria were found between the regions, with indications of winter peaks in both regions but a spring peak in the NW. The presence of Campylobacter bacteria on boot socks was negatively associated with ambient temperature (*P* = 0.011) and positively associated with precipitation (*P* < 0.001), results consistent with our understanding of Campylobacter survival and the probability of material adhering to boot socks. Campylobacter jejuni was the predominant species found; Campylobacter coli was largely restricted to the livestock-dominated NW. Source attribution analysis indicated that the potential source of C. jejuni was predominantly sheep in the NW and wild birds in EA but did not differ between peak and nonpeak periods of human incidence.

**IMPORTANCE** There is debate in the literature on the pathways through which pathogens are transferred from the environment to humans. We report on the success of a novel method for sampling human-pathogen interactions using boot socks and citizen science techniques, which enable us to sample human-pathogen interactions that may occur through visits to natural environments. This contrasts with traditional environmental sampling, which is based on spot sampling techniques and does not sample human-pathogen interactions. Our methods are of practical value to scientists trying to understand the transmission of pathogens from the environment to people. Our findings provide insight into the risk of Campylobacter exposure from recreational visits and an understanding of seasonal differences in risk and the factors behind these patterns. We highlight the Campylobacter species predominantly encountered and the potential sources of C. jejuni.

## INTRODUCTION

Campylobacter species are the most common bacterial causes of diarrheal disease in the developed world (1). While most cases are self-limiting, longer-term sequelae include irritable bowel syndrome and Guillain-Barré syndrome (2). In the European Union, Campylobacter infection is the most frequently reported foodborne illness, estimated to cause more than 190,000 human cases annually. Due to underreporting, the true incidence is believed to be around 9 million annually. The cost of Campylobacter infections to public health systems and the cost in lost productivity in the European Union are estimated at EUR 2.4 billion a year (3). In England and Wales, more than 1 million cases have been recorded officially since the emergence of such infections in the 1970s (1), and given the underreporting that is known to occur, this equates to more than 10 million cases in total (4).

Campylobacter epidemiology is complex; one of the most prominent features is a strong seasonal peak in cases. This occurs toward the end of June in the United Kingdom (5). The causes of this seasonal peak are unknown. Proposed hypotheses include changes in food preparation (e.g., barbecues), increases in chicken flock colonization, and seasonal changes in human engagement with the outdoor environment, in which Campylobacter species might be present (6). Human Campylobacter infections have also been shown to be positively associated with temperature (5) and precipitation (7), although the precise mechanisms for these associations are unclear. Within England, there are distinct regional differences in cases: greater numbers of cases are seen in northern and western regions than in southern and eastern England (1, 5). Although foreign travel is associated with 20% of Campylobacter cases in the United Kingdom (8), the peak in travel-associated cases occurs later in the summer (9). Conventionally it is assumed that most Campylobacter cases are transmitted from food, primarily poultry meat. However, recent studies estimate that foodborne routes of exposure account for approximately 50% of the disease burden (10, 11). Therefore, nonfood pathways of transmission are likely to be important for Campylobacter infection.

The predominant human pathogens (Campylobacter jejuni and, to a lesser extent, Campylobacter coli) are found in ruminants, pigs, poultry, and wild birds and animals (12). Furthermore, studies indicate that high Campylobacter levels are found in individual environmental components, such as soils, feces, and water (13). Recreational visits to the countryside have been hypothesized to be one pathway through which Campylobacter in the environment could be transferred to people (6). People could pick up material containing Campylobacter species as they walk through these environments as part of outdoor recreation via (for example) their shoes, their hands touching fences and the ground, or splashes from mud onto clothes. This material could ultimately be transferred into their mouths, facilitating the transfer of Campylobacter to humans.

To investigate this possibility, we deployed a novel method for sampling human-environment interactions, specifically, the use of fabric boot socks worn over citizen scientists' boots. This has advantages over traditional methods of environmental sampling, such as small-site samples of water or soil, in that we obtain a composite sample from a large geographical area. Most importantly, however, this method samples the human-environment interaction and indicates whether after such an interaction, in this case a countryside walk, the individual's footwear is positive or negative for Campylobacter. The method also samples the entire foot, unlike some previous studies that have taken footwear swabs (14). Boot sock sampling methods have been used before in small-scale farm sampling (e.g., broiler houses [15]), but here we extend their use in order to examine the method's utility for outdoor environmental sampling over far greater distances (up to 4 km) and for longer durations (ca. 60 min). This study is also novel in that we utilize citizen science techniques (16), in which citizens participate in the collection of microbiological data. Specifically, we utilized networks of volunteer walkers to undertake the environmental sampling in 40 individual weeks over a 16-month period.

In this paper, we present our methodology for using citizen scientists to sample human-environment interactions using boot socks. We then validate this methodology by considering the utility of citizen scientists for environmental sampling and examine the internal consistency of our method by comparing the results from multiple walkers traversing the same route in the environment. We show that our methods are able to detect Campylobacter on walkers' shoes and highlight the Campylobacter species found. We also attribute the C. jejuni strains found to potential sources through source attribution using microbial subtyping. We present results on the seasonality of Campylobacter-positive boot socks in two contrasting regions of England and subsequently generate statistical models to examine the impact of a variety of environmental variables (e.g., temperature, rainfall) on the probability of Campylobacter-positive boot socks.

## RESULTS

### Methodological validation.

Our experience of using “citizen scientists” in the form of networks of walkers is overwhelmingly positive. Altogether, 60 people volunteered to participate in the study. They were enthusiastic in their training and willing to take part in the study. Demographically they were a mix of genders and ranged in age from 18 to 80 years. Training was simple, and the walkers engaged fully with the study. This extended to submitting photos, text, and even a poem to the research team for use in quarterly newsletters. In terms of science delivery, all 240 walks were successfully undertaken, and all 720 boot socks were mailed to the laboratory on the day of the walk. For all walks, observations of livestock, underfoot conditions, and weather were successfully made. Variable mobile data signals in several of the locations meant that some walkers recorded these data on paper forms and uploaded them later in the day, rather than using the smartphone. This did not affect the global positioning system (GPS) recordings for the walks, which were automatically recorded and uploaded.

The livestock seen by walkers on the different walk routes did not always reflect the livestock expected from the agricultural survey. In particular, in the Lancashire foothills walk, sheep rather than cows were seen. In Suffolk, no livestock were seen. Pigs were seen just once in each region.

All 720 boot socks (6 walking routes × 40 weeks × 3 boot socks) were received at the laboratory for analysis within 72 h of the walk. Overall, 94% arrived within 24 h; 5% arrived between 24 and 48 h after the walk; and just 1% arrived between 48 and 72 h. After each walk, meetings were held between the walker management and the laboratory teams to confirm that each walk had successfully taken place and to identify any sampling issues to which the walkers could be alerted. The most common of these was failure to place the absorbent sachet in the second bag; occasionally, walkers did not place the boot sock in the biohazard bag.

The method proved capable of detecting Campylobacter bacteria on boot socks. In this study, boot socks were classified as positive if they were found positive by either PCR or culture. Of the 240 walks undertaken, 156 (65.0%) resulted in at least one boot sock testing positive for Campylobacter either by culture or by PCR. Table 1 shows the total number of boot socks that tested positive and how this differed by region. Overall, 340 (47.2%) boot socks were positive for Campylobacter spp. by either culture or PCR; 55.8% of boot socks from the North West (NW) and 38.6% of boot socks from East Anglia (EA) were positive (*P*, <0.001 by Fisher's exact test for the comparison between the NW and EA). Table 1 also highlights the numbers of boot socks found to be positive by the two different test methods (culture and PCR). Almost all the positive NW boot socks were found to be positive by PCR (89.1%) and culture (85.6%). The positive boot socks from EA were mostly found to be positive by PCR (93.5%); only 54.7% were found positive using culture (no significant difference in PCR positivity was found between the NW and EA; *P*, <0.001 for the difference in culture-positive results).

**TABLE 1:** Total positive boot socks found using PCR and culture methods by region

Region	No. (% [95% CI]) of all boot socks positive for Campylobacter^*a*^	No. (% [95% CI] of all Campylobacter-positive boot socks) of boot socks found positive by the following method:			
		PCR	PCR only	Culture	Culture only
NW	201 (55.8 [50.7–61.0])*	179 (89.1 [84.7–93.4])	29 (14.4 [9.6–19.3])	172 (85.6 [80.7–90.4])	22 (10.9 [6.6–15.3])
EA	139 (38.6 [33.6–43.6])*	130 (93.5 [89.4–97.6])	63 (45.3 [37.0–53.6])	76 (54.7 [46.4–63.0])	9 (6.5 [2.4–10.6])
Total	340 (47.2 [43.6–50.9])	309 (90.9 [87.8–93.9])	92 (27.0 [22.3–31.8])	248 (72.9 [68.2–77.7])	31 (9.1 [6.1–12.2])

a In total, 720 boot socks were analyzed: 360 in the NW and 360 in EA. 95% CI, 95% confidence interval. Asterisks indicate significant differences (*P* < 0.001) by Fisher's exact test comparing the proportions of positive boot socks in the NW and EA.

**FIG 1:**
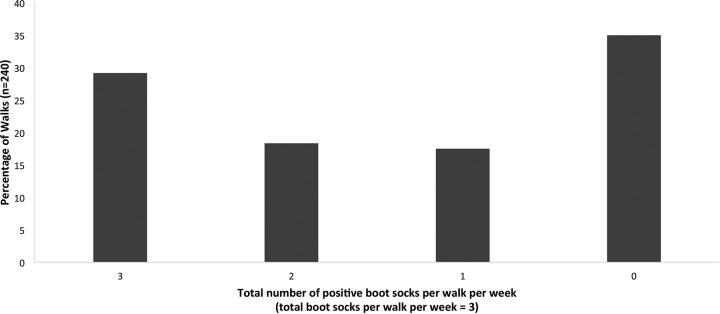
Percentages of walks with 0, 1, 2, and 3 positive boot socks as a measure of the internal consistency of walks.

To validate the boot sock as a method for environmental sampling, we examined the consistency of the boot socks from each walk with each other. All 240 walks had three boot socks (from three individual walkers). An internally consistent walk was defined as one where all three boot socks showed the same result; i.e., all three boot socks were either positive or negative for Campylobacter (by culture or PCR). The internal consistency of walks is shown in Fig. 1. Of the 240 walks, 70 (29.2%) had three positive boot socks and 84 (35.0%) had three negative boot socks. If the walks had not been internally consistent, then in terms of probability, we would have expected a clear majority of walks to exhibit mixtures of positive and negative boot socks. This was not observed, providing evidence of internal consistency.

The Campylobacter species found on the boot socks in the two regions are shown in Table 2. Species were identified only on culture-positive boot socks (*n* = 248). Table 2 shows that the dominant species in both regions was C. jejuni (62.2% in the NW; 84.2% in EA), while almost all C. coli-positive boot socks were found in the NW (27.4% in the NW; 2.6% in EA). This difference in species between regions was statistically significant (*P* < 0.001).

**TABLE 2:** Species of Campylobacter identified from culture-positive boot socks

Region	No. (% [95% CI]) of boot socks found positive for Campylobacter by culture	No. (% [95% CI] of all culture-positive boot socks) of boot socks^*a*^ positive for:				
		C. jejuni	C. coli	Mixed C. jejuni and C. coli	Other species	Species not determined^*b*^
NW	172 (85.6 [80.7–90.4])	107 (62.2 [55.0–69.5])*	47 (27.4 [20.7–34.0])*	16 (9.3 [5.0–13.6])	2 (1.2 [−0.4–2.8])	0
EA	76 (54.7 [46.4–63.0])	64 (84.2 [76.0–92.4])*	2 (2.6 [−1.0–6.2])*	2 (2.6 [−1.0–6.2])	7 (9.2 [2.7–15.7])	1 (1.3 [−1.2–3.9])
Total	248 (72.9 [68.2–77.7])	171 (69.0 [63.2–74.7])	49 (19.8 [14.8–24.7])	18 (7.3 [4.0–10.5])	9 (3.6 [1.3–5.6])	1 (0.4 [−0.4–1.2])

a Asterisks indicate significant differences (*P* < 0.001) by Fisher's exact test comparing C. jejuni and C. coli in the NW and EA. 95% CI, 95% confidence interval.

b Campylobacter bacteria died before species identification.

**FIG 2:**
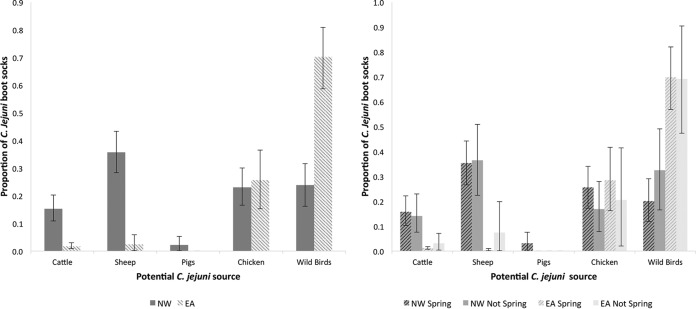
Proportions of boot socks positive for C. jejuni attributed to potential sources of C. jejuni by region alone (left) and by region and season (right), with 95% bootstrap confidence intervals.

The graph on the left of Fig. 2 shows the potential source attribution of the C. jejuni bacteria found in each region. In EA, the dominant potential source of C. jejuni was wild birds (70%), while in the NW, the sources were more mixed. Sheep were the largest potential source in the NW (36%), while wild birds and retail chicken each accounted for approximately 25% of isolates. The graph on the right of Fig. 2 shows the potential source attribution of the C. jejuni bacteria found in each region during the spring peak (April to July) and at other times of the year. Within regions, potential source attributions were similar at different times of the year.

**FIG 3:**
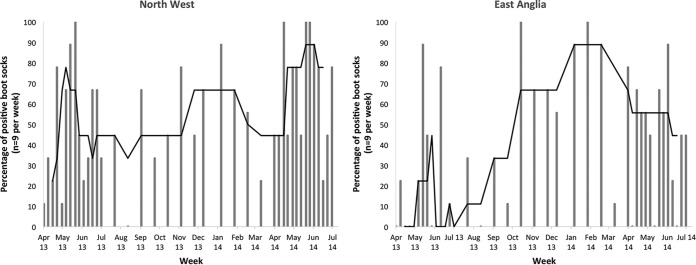
Differences in the percentages of positive boot socks each week across the study period, by region. Each bar indicates the percentage of boot socks that were positive for Campylobacter in a particular week (*n* = 9). The line indicates the running median over five sample weeks.

In terms of differences in the proportion of positive boot socks over the study period, Fig. 3 shows the percentage of boot socks that tested Campylobacter positive each week in the NW and EA. The line on each graph shows the 5-week (the current week and 2 sampling weeks on either side) running median. Care must be taken in interpreting these data, due to small sample sizes and interweek variability. However, the figure is suggestive of peaks in both spring periods and in the winter in the NW region. In EA, the highest levels of positive boot socks were found in the winter months, with little consistent evidence for a peak in spring across the two spring periods.

A mixed-effects logistic model was run to determine the associations between positive boot socks and the environmental conditions in the walk area. The results are presented in Table 3. The week and route of the walk were included as random effects in the model. Significant associations with temperature and rainfall were found (Wald χ^2^ = 24.61; *P* > χ^2^ < 0.001). The strongest associations were found for the environmental conditions in the 7 days prior to the walk date. The average (over 7 days) maximum morning air temperature was negatively associated with positive boot socks, while the average (over 7 days) daily rainfall had a positive but nonlinear association, as indicated by the inclusion of the quadratic term. The quadratic term was included rather than a logged predictor because the relationship between positive boot socks and rainfall was not monotonic. In these final models, river flow and the variables collected by the walkers were insignificant. Examination of the model deviance residuals shows that they were normally distributed, and a plot of the deviance residuals against the fitted values produced straight lines, signifying an appropriate model structure (see Fig. S1 and S2 in the supplemental material).

**TABLE 3:** Mixed-effects logistic model showing the influence of the previous 7 days' temperature and rainfall on boot sock positivity^*a*^

Variable in the route area in the 7 days prior to the walk	Odds ratio (95% CI)	SE	*P*
Avg maximum morning air temp (°C)	0.87 (0.79–0.97)	0.05	0.011
Avg daily rainfall (mm)	2.99 (1.83–4.88)	0.74	<0.001
Avg daily rainfall squared (mm)	0.90 (0.85–0.95)	0.03	<0.001

a Wald χ^2^ = 24.61 (*P* > χ^2^ < 0.001). The week and the route of the walk were included as random effects. 95% CI, 95% confidence interval.

## DISCUSSION

In this study, we present a novel methodology utilizing boot socks and citizen scientists to evaluate human-pathogen interactions. Working with citizens requires significant inputs in terms of delivering appropriate training and providing regular support and feedback. However, if these are in place, citizen science can be used for successful, independent, long-term, and systematic environmental sampling. All walks were successfully completed, every boot sock mailed to the laboratory, and all additional observations submitted.

Almost half of the boot socks (47.2%) worn on a 4-km walk in the countryside were positive for Campylobacter either by PCR or by culture. Previous studies using boot socks have done so in more-controlled environments, such as poultry sheds (15), or have swab-sampled shoes rather than sampling whole footwear (14). We demonstrate that by using boot socks with citizen scientists, on walks that were substantial in distance, and sending boot socks to the laboratory through the postal system, we were able to extract Campylobacter bacteria from a high proportion of boot socks. This highlights the potential of spatially distributed, citizen science-based environmental sampling (17) for pathogens or, potentially, for other particles in the environment.

Here we present data for boot socks positive by either PCR or culture. The ability to culture Campylobacter or to detect it by PCR is affected by the loading of Campylobacter in the environment. Campylobacter can be detected by these methods only if a high enough level of the pathogen is present. PCR is the more-sensitive method, and more of our boot socks were positive by PCR than by culture (90.9% versus 72.9%). When a sample is positive by PCR only, the finding suggests that the bacteria may be present in low numbers, dead, stressed, or in what is known as a viable but nonculturable (VBNC) state. In the NW, similar percentages of boot socks were found positive by the PCR and culture methods. In EA, far more boot socks were positive by PCR than by culture. This suggests that the Campylobacter bacteria found in EA may be present only in low numbers or may be in a VBNC state. There is, however, some evidence that bacteria in a VBNC state can still be infectious to humans and pose a public health risk (18). Some boot socks were found to be positive by culture but negative by PCR. This could be due to a carryover of potential inhibitors during the Chelex extraction that inhibited the PCR assay, an unusual situation but one that can occur when there are low numbers of pathogen.

Our confidence in using boot socks as a method for sampling the environment is enhanced by the evidence of consistency among the three boot socks worn on each walk. On 64% of the walks, all three boot socks produced the same result. Our methodological confidence is enhanced by the adoption of this approach by Public Health England to sample for the presence of Escherichia coli O55 in the environment. This was done during an outbreak in Dorset in the summer of 2015, and the research team was contacted based on a presentation at a national Campylobacter conference (19). This approach involved public health officials rather than citizen scientists, and the method was adapted to be used for hands as well as feet.

C. jejuni was the predominant species found in the environment (∼70%); C. coli was found at lower frequencies (∼20%). This result supports other studies that have shown C. jejuni to be the predominant species found in animal feces (13). The majority of C. coli bacteria were found in the NW region. This finding may reflect the high numbers of sheep present in this region (20), as observed by our walkers in the NW. C. coli is found commonly in sheep feces (13). It is also present in pigs (21), but these were rarely seen.

The source attribution element of the study showed that the potential sources of C. jejuni in the environment differ considerably between the regions. However, within each region, the results do seem to match the likely potential sources of exposure based on the region's agricultural characteristics. In EA, wild birds were the main potential source of C. jejuni (71%), a finding that likely reflects the low levels of livestock in EA and hence the likely predominance of wild bird sources. In the NW, however, only 25% of C. jejuni isolates were potentially derived from wild birds; sheep (32%) were the main potential source of C. jejuni. This is not unexpected, since sheep are abundant in the NW and were commonly observed on all three NW routes. There was no evidence that the potential sources of C. jejuni in the environment differed between peak and nonpeak periods of human incidence.

Interpretation of the differences in our results between the spring and winter periods needs to be cautious, because sampling occurred only in two spring seasons and one winter season, sample numbers were not large, and there was variability in the numbers of positive boot socks from week to week. However, our findings show some evidence of a spring peak in the NW, with generally higher Campylobacter levels in the months of May and June than in other months in both years. This finding is interesting, given the recognized seasonal peak in human Campylobacter cases in the United Kingdom (1, 5). However, in EA, there was no suggestion of a peak in the spring months. This is also interesting because in this region, the spring peak in human Campylobacter cases is less distinct than that in the NW (1, 5).

In EA and, to a lesser degree, in the NW, there was a suggestion of a peak in Campylobacter-positive boot socks during the winter months. This may indicate that during these cool winter months, Campylobacter survival is high, and the damp conditions are ideal for enabling material to adhere to the boot sock. It is not clear why this pattern is less pronounced in the NW, but the difference may possibly be associated with lower overall rainfall amounts in EA, leading to less dilution. No winter peak in human Campylobacter cases is observed, but at this time of year, visits to the countryside are typically low.

An understanding of these patterns is enhanced by the multivariate analysis of Campylobacter-positive boot socks. In interpreting these results, it is important to recognize that a positive boot sock result depends on the presence of Campylobacter in the environment and the adherence of positive material to the boot sock. Our finding that the presence of Campylobacter on boot socks is associated with increased rainfall over the previous 7 days supports results from other studies indicating that Campylobacter survives better under damp conditions (22–24). However, this finding may also indicate that damp conditions enhance soil moisture and hence increase the probability of material adhering to the boot sock. The quadratic relationship with rainfall could result from higher water levels flushing Campylobacter from the system or a dilution effect (25, 26). The statistical analysis also indicated that Campylobacter-positive boot socks were negatively associated with ambient temperature. This is consistent with studies indicating enhanced Campylobacter survival at low temperatures (27, 28).

There are limitations in using boot socks for environmental sampling; one is that they produce a composite environmental sample, and it is not possible to determine precisely where Campylobacter was encountered. It would be possible to use multiple boot socks over smaller sections of each walk, although this requirement may present challenges for citizen scientists. While our method presents issues for environmental sampling, it is ideal for sampling human-environment interactions. It is not known how the risk explored in this study using boot socks relates to the risk of a walker using a shoe or walking boot with features such as treads and laces, although a previous study where shoes were swabbed suggests that deep treads enhanced the adhesion of pathogens to shoes (14). Further work is needed to relate the presence of Campylobacter bacteria on footwear to disease risk in humans. Another limitation of the use of boot socks is that it provides results in terms of presence or absence; there is no measure of quantity or “loading.”

To conclude, we present a novel methodology for assessing human-pathogen interactions in the natural environment using boot socks and citizen scientists. Our use of networks of volunteer walkers wearing boot socks was shown to be a successful data collection method and produced boot socks from which Campylobacter bacteria were successfully extracted. Confidence in our results is enhanced through the observation that when multiple boot socks were worn on the same walk, there was a high degree of internal consistency.

The boot sock method using citizen scientists can provide important insights into the presence of Campylobacter in the environment and its implications for human health. Boot socks measure potential human-pathogen interactions, and the results indicated that just under half of all boot socks were positive for Campylobacter either by culture or by PCR after a 4-km walk in the countryside. C. jejuni was the Campylobacter species predominantly found in the environment, while C. coli was largely restricted to the livestock-dominated NW. Source attribution modeling indicated that the C. jejuni strains matched the regional livestock characteristics, with wild bird sources providing an important component, especially in EA. There was no indication from this modeling that the source differed between the spring peak in human cases and other times of the year. We highlighted the seasonal difference in Campylobacter presence on boot socks and indicate a spring peak in positivity in the livestock-dominated NW but not in EA. This peak coincides with the spring peak in human cases. Both regions show a peak in the winter, but at a time when outdoor recreation is limited. Campylobacter-positive boot socks show significant associations with decreasing temperature and increased rainfall, results consistent with our understanding of Campylobacter survival and the probability of material adhering to boot socks.

## MATERIALS AND METHODS

This study was conducted in two English regions, the North West (NW) and East Anglia (EA), which have different Campylobacter infection rates. In the NW, the seasonal peak in human Campylobacter infection is more pronounced than in EA. The agricultural characteristics and climates of the two regions are also markedly different: the NW is wetter than EA and is dominated by livestock, while EA is drier and mostly arable.

Within EA and the NW, candidate walking routes were selected. To ensure that these were frequented by walkers, areas in the top quintile of visits within each region were selected. This was achieved using a gridded density map of visits to natural areas (29).

To ensure that these areas were regionally representative in terms of likely Campylobacter sources, candidate sites that had numbers of livestock (sheep, cows, or cows and sheep combined) within the median regional quintile were identified. These data were obtained from the UK Agricultural Census, which provides data on the numbers of livestock in kilometer grid squares (20). Areas satisfying both these criteria and measuring 4 km^2^ (to provide a large walk area) were retained. Within or near these areas, large-scale maps (OS 1:25,000) (30) were used to identify circular 4-km walking routes (to keep the time and cost requirements of sampling manageable) along public rights of way. Routes had to have an accessible starting point. Each potential walk was inspected by the research team to ensure that it was mostly on natural surfaces (e.g., soil or grass) and that the route was safe in all kinds of weather and was not proximate to a likely Campylobacter hot spot (e.g., a large poultry farm), in order to avoid skewed data. Routes did not include any long sections (>100 m) across arable fields, because during trials, excessive amounts of soil from newly plowed fields caused the boot sock to detach. Three routes that were spatially dispersed (at least 30 km apart) and that included the various regional landscapes were selected in each region (i.e., six in total). The six routes were as follows: (i) NW uplands, Cumbria (predominantly sheep); (ii) NW foothills, Lancashire (predominantly cattle); (iii) NW lowlands, Cheshire (predominantly cattle and sheep); (iv) EA lowlands, Norfolk (predominantly arable land, cattle, and sheep); (v) EA lowlands, Suffolk (predominantly arable land, cattle); (vi) EA lowlands, Norfolk (predominantly arable land interspersed with wetlands, sheep).

The six routes were walked weekly from April 2013 to July 2013 and from April 2014 to July 2014. Between these two periods, walks were conducted every 3 weeks. This frequency ensured that sampling was intensified around the seasonal Campylobacter peak. In total, walks were undertaken on 40 separate weeks on each of the 6 walking routes (i.e., 240 walks in total). Each of the walks was traversed by 3 individuals walking together, resulting in 3 samples for each walk and a total of 720 boot socks across the whole study. Three walkers were chosen so that replicates would be produced for each walk (i.e., 3 walkers on each of the 6 walking routes on every week when walks occurred).

A boot sock protocol was developed to ensure correct use by walkers. At the start of each walk, each walker placed a plastic overshoe over the shoe on his or her leading foot to prevent contamination between the shoe and the boot sock. A new boot sock (60-g disposable shoe cover; Tunika) was placed over the plastic overshoe. The walkers then traversed the specified route and were asked to walk normally. During the walk, the participants were asked to check their boot socks for large tears. If any were found, another boot sock was placed over the existing boot sock. At the end of the walk, participants removed their boot socks using clean gloves. Each individual walker then placed his or her boot sock(s) in an individual biohazard specimen bag, which was sealed and labeled using a precompleted laboratory sample form. The biohazard bag was placed inside a second sealable plastic bag to prevent leakage, and an absorbent sachet was added to the second bag to remove excess moisture. This plastic bag was then placed in a preaddressed plastic envelope, which specified (to conform to postal regulations) that it contained a UN3373 Biological Substances diagnostic specimen, and was mailed to the Preston Public Health England Laboratory for the first month of the study, and subsequently to the University of Liverpool laboratories, to be tested for the presence of Campylobacter. Studies have indicated that Campylobacter bacteria can survive for as long as 2 weeks at ambient temperatures (31), and thus, the postal service was deemed an appropriate method for transporting boot socks.

Walkers were recruited from local walking groups and through advertisements placed close to the selected routes. Written consent to take part in the study was obtained (UEA consent form 150113). Walkers were screened using a health questionnaire to identify those able to walk the route safely. As already mentioned, each walk was traversed by three individuals walking together for both replication and safety. In order to prevent possible contamination, walking with pets was not allowed. All walks were undertaken at similar times each week (Mondays from 10 a.m. to 2 p.m.). Walkers were offered a small remuneration in the form of supermarket vouchers for each completed walk. A training event, including a pilot walk, was held to explain the boot sock protocol, and walkers were provided with written instructions and guidance on how to deal with any eventuality. An emergency phone was supplied.

A subset of walkers were designated walk leaders and were provided with extra training regarding the correct procedure for collecting and preparing the boot socks and mailing them to the laboratory. One walk leader was present on each walk. Walkers were sent a reminder (by text, phone, or email) by the research team in advance of each walk. Members of the research team periodically accompanied the walkers to ensure that the protocol was adhered to. To maintain engagement with such a large group of walkers, a newsletter about the project was produced quarterly and was sent to all walkers.

To further characterize each walk, walkers were asked to complete an observational survey on each walk. For this purpose, each walk leader was provided with a smartphone that was used to collect and submit observational data. The route was divided into three sections (contiguous parcels of visible land), and for each walk, walkers were asked to categorize the number (0, 0 to 10, >10) and type of livestock (sheep, cows, horses, and pigs) seen in each section. They were also asked to record the weather and the underfoot conditions. Additionally, the route taken during the walk was recorded using a GPS tracking application. This was automatically uploaded to the research team and provided validation that the walk had been successfully completed.

At the laboratory, multiple boot socks from one walker (i.e., where large tears had developed in the boot sock during the walk, and a second boot sock had been placed over the first) were pooled to produce one result per walker (referred to here as a positive or negative “boot sock” [singular]). Therefore, 3 boot sock samples were analyzed for each walk, resulting in 720 boot socks (6 walking routes × 40 weeks × 3 boot socks). Upon receipt in the laboratory, boot sock details were logged, and 100 ml of buffered peptone water (BPW) was added to each boot sock and hand palpated for 1 min. Boot socks were left for 10 min to allow the sediment to settle, and 10 ml of the supernatant was then added to 10 ml of double-concentrated Exeter broth (1.1 liter nutrient broth, 11 ml lysed defibrinated horse blood, Campylobacter enrichment supplement SV59 [Mast Group Ltd., Bootle, United Kingdom], and Campylobacter growth supplement SV61 [Mast Group Ltd.]). This mixture was incubated under microaerobic conditions (80% N_2_, 12% CO_2_, 5% O_2_, and 3% H_2_) in a variable-atmosphere incubator (Don Whitley Scientific Ltd., Shipley, United Kingdom) at 41 ± 1°C for 48 h.

A 47-mm-diameter, 0.45-μm-pore-size cellulose nitrate filter (Sartorius, Epsom, United Kingdom) was placed on the surface of a modified charcoal cefoperazone deoxycholate agar (mCCDA) Campylobacter selective plate containing cefoperazone (32 mg/liter) and amphotericin B (10 mg/liter). Altogether, 100 μl of enriched broth was added to the agar plate and was then spread over the surface of the filter and allowed to sit at room temperature for 30 min. After this time, the filter was removed, and the plates were incubated as described above for as long as 72 h. The addition of the filter aided in the recovery of Campylobacter species from the broth, since, due to their size and mobility, they are able to pass easily through the filter onto the agar plate surface, while other, contaminating bacteria are retained on the filter surface. Incubation plates were examined for the presence of Campylobacter spp., and as many as four suspect Campylobacter colonies were subcultured on two Columbia blood agar plates containing 5% defibrinated horse blood. One plate was incubated under microaerobic conditions for 48 h at 41°C and the other under aerobic conditions at 30°C for 48 h. Isolates that grew under microaerobic conditions only were retained for further study. All media and supplements were from Lab M (Bury, United Kingdom) unless stated otherwise, and blood was obtained from Southern Group Labs Ltd. (Corby, United Kingdom).

Cell lysates for PCR identification were prepared from fresh cultures using a Chelex method (32). Briefly, a loopful of bacteria was suspended in 300 ml of a 20% (wt/vol) Chelex solution (Chelex-100 in 10 mM Tris-HCl and 1 mM EDTA [pH 8]) (Bio-Rad, United Kingdom), and the suspension was heated at 95°C for 10 min and was then centrifuged at 10,000 rpm for 2 min. Fifty microliters of the supernatant added to 450 μl of sterile deionized water was used for PCR amplification. A genus-specific PCR assay was used to confirm an isolate as a member of a Campylobacter species as described previously (33). For each sample found to contain a Campylobacter species, one isolate was subjected to an *lpx* gene PCR assay to determine if it was C. jejuni or C. coli. If it tested negative for these species, screening for Campylobacter upsaliensis or Campylobacter *lari* was undertaken (34). Isolates not assigned to any of these species were further screened using Campylobacter hyointestinalis and Campylobacter fetus PCR assays (35). Isolates still not assigned to a species were subjected to amplification of the *groEL* gene followed by Sanger sequencing as described previously (36, 37). All PCR reagents were obtained from Solis BioDyne, Estonia.

One milliliter of the enriched broth samples also underwent the DNA Chelex extraction method as described above and was subjected to the PCR assay described by Katzav et al. (33) to determine whether the samples were positive for Campylobacter species. Therefore, even if no positive boot socks were found during the culture process, PCR analysis was still undertaken on the boot socks. This meant that boot socks could have been found to be positive by culture only, by PCR only, or by both methods.

Further analysis was undertaken on two-thirds of the boot socks positive for Campylobacter by culture, with a single isolate of C. jejuni selected per boot sock walk for whole-genome sequencing. TruSeq Nano DNA Illumina libraries were prepared for each DNA sample and were subsequently sequenced on the Illumina HiSeq 2500 or 4000 platform to generate 125-bp paired-end reads. Reads were trimmed to remove low-quality bases and sequencing adaptors using Sickle v1.200 (38) and Cutadapt v1.2.1 (39), respectively. Each set of reads was *de novo* assembled into scaffolds using SPAdes v3.7.0 (40). These scaffolds were interrogated to determine the multilocus sequence typing (MLST) alleles and sequence type for each isolate. This was achieved by aligning MLST allele sequences obtained from the PubMLST website (pubmlst.org) against each genome assembly using Bowtie2 (41). For each locus, if an allele sequence aligned perfectly, the sample was assigned this allele. If novel alleles were detected (i.e., the best alignment for a locus was a nonperfect but full-length match), they were submitted to pubmlst.org.

Structure software (42) was used to assign boot sock MLST isolate data to one of five potential infection sources (cattle, sheep, pigs, wild birds [all fecal isolates]), and chicken [all retail food isolates]). This was achieved by determining the genotype frequencies among each of the potential infection sources; the uncertainty regarding this determination of genotype frequency resulted from the probabilistic rather than absolute nature of the attribution scores for each isolate (using the method of Sheppard et al. [11]). The average proportion attributed by source was calculated, and the corresponding 95% confidence intervals (CIs) (percentiles) were calculated using 10,000 Monte Carlo steps (43).

To characterize each walk further, data on environmental conditions on the day of the walk and for a period of 28 days previously were obtained. These environmental variables were hypothesized to be related to the presence of Campylobacter bacteria on boot socks. The maximum morning temperature (in degrees Celsius) for the six routes was obtained from the Met Office Integrated Data Archive System (MIDAS) Land and Marine Surface Stations data set (44). These data were from the Automatic Weather Station nearest (1 to 37 km) to the route with complete data for the time period required. Using the same data source, daily rainfall (in millimeters) from the Water Authority station nearest (2.5 to 8 km) to the route was obtained for the time period. As a measure of soil moisture (which may relate to periods when material is more likely to adhere to the boot sock), river flow data were obtained for the six different walk locations from the National River Flow Archive (45). The nearest (1.5 to 9.5 km) gauging station for the river catchment within which each walk was located in was determined, and the gauged daily flow (in cubic meters per second) for the study period was obtained. Measured soil moisture was obtained for two locations from the UK Environmental Change Network. However, because these values were not specific to individual routes, they could not be used. For similar reasons, one measurement of UV radiation, obtained from the Department for Food and Rural Affairs, was excluded.

Temperature, rainfall, and river flow for the day previous to the walk and averages for the 7 days and the 28 days prior to the walk were calculated. These data on environmental conditions were combined with the data collected from the citizen scientists (livestock observed, weather, and underfoot conditions) and were used as explanatory variables for positive boot socks. All analysis was undertaken using STATA 11, using a mixed-effects logistic regression model with the route and week as random effects.

The study received ethical clearance from the Faculty of Medicine and Health Science Research Ethics Committee at the University of East Anglia (2012/2013-40).

### Accession number(s).

The raw sequence data were deposited in the EBI ENA database (http://www.ebi.ac.uk/ena) with accession no. PRJEB20152.

## Supplementary Material

Supplemental material

## References

[1] Nichols GL, Richardson JF, Sheppard SK, Lane C, Sarran C 2012 Campylobacter epidemiology: a descriptive study reviewing 1 million cases in England and Wales between 1989 and 2011. BMJ Open 2:e001179. doi:10.1136/bmjopen-2012-001179. PMC3400078 22798256

[2] Keithlin J, Sargeant J, Thomas MK, Fazil A 2014 Systematic review and meta-analysis of the proportion of Campylobacter cases that develop chronic sequelae. BMC Public Health 14:1203. doi:10.1186/1471-2458-14-1203. 25416162 PMC4391665

[3] EFSA Panel on Biological Hazards. 2011 Scientific Opinion on Campylobacter in broiler meat production: control options and performance objectives and/or targets at different stages of the food chain. EFSA J 9:141.

[4] Tam CC, Rodrigues LC, Viviani L, Dodds JP, Evans MR, Hunter PR, Gray JJ, Letley LH, Rait G, Tompkins DS, O'Brien SJ 2012 Longitudinal study of infectious intestinal disease in the UK (IID2 study): incidence in the community and presenting to general practice. Gut 61:69–77. doi:10.1136/gut.2011.238386. 21708822 PMC3230829

[5] Louis VR, Gillespie IA, O'Brien SJ, Russek-Cohen E, Pearson AD, Colwell RR 2005 Temperature-driven Campylobacter seasonality in England and Wales. Appl Environ Microbiol 71:85–92. doi:10.1128/AEM.71.1.85-92.2005. 15640174 PMC544220

[6] Nichols GL 2005 Fly transmission of Campylobacter. Emerg Infect Dis 11:361–364. doi:10.3201/eid1103.040460. 15757548 PMC3298251

[7] Nichols G, Lane C, Asgari N, Verlander NQ, Charlett A 2009 Rainfall and outbreaks of drinking water related disease in England and Wales. J Water Health 7:1–8. doi:10.2166/wh.2009.143. 18957770

[8] Gillespie IA, O'Brien SJ, Penman C, Tompkins D, Cowden J, Humphrey TJ 2008 Demographic determinants for Campylobacter infection in England and Wales: implications for future epidemiological studies. Epidemiol Infect 136:1717–1725. doi:10.1017/S0950268808000319. 19000328 PMC2870783

[9] Strachan NJC, Rotariu O, Smith-Palmer A, Cowden J, Sheppard SK, O'Brien SJ, Maiden MCJ, MacRae M, Bessell PR, Matthews L, Reid SWJ, Innocent GT, Ogden ID, Forbes KJ 2013 Identifying the seasonal origins of human campylobacteriosis. Epidemiol Infect 141:1267–1275. doi:10.1017/S0950268812002063. 22989449 PMC4003528

[10] Davis MA, Moore DL, Baker KNK, French NP, Patnode M, Hensley J, MacDonald K, Besser TE 2013 Risk factors for campylobacteriosis in two Washington State counties with high numbers of dairy farms. J Clin Microbiol 51:3921–3927. doi:10.1128/JCM.01433-13. 24025908 PMC3838072

[11] Sheppard SK, Dallas JF, Strachan NJC, MacRae M, McCarthy ND, Wilson DJ, Gormley FJ, Falush D, Ogden LD, Maiden MCJ, Forbes KJ 2009 Campylobacter genotyping to determine the source of human infection. Clin Infect Dis 48:1072–1078. doi:10.1086/597402. 19275496 PMC3988352

[12] Jones K 2001 Campylobacters in water, sewage and the environment. J Appl Microbiol 90(S6):68S–79S. doi:10.1046/j.1365-2672.2001.01355.x. 11422562

[13] Brown PE, Christensen OF, Clough HE, Diggle PJ, Hart CA, Hazel S, Kemp R, Leatherbarrow AJH, Moore A, Sutherst J, Turner J, Williams NJ, Wright EJ, French NP 2004 Frequency and spatial distribution of environmental Campylobacter spp. Appl Environ Microbiol 70:6501–6511. doi:10.1128/AEM.70.11.6501-6511.2004. 15528512 PMC525266

[14] Rashid T, VonVille HM, Hasan I, Garey KW 2016 Shoe soles as a potential vector for pathogen transmission: a systematic review. J Appl Microbiol 121:1223–1231. doi:10.1111/jam.13250. 27495010

[15] Berghaus RD, Thayer SG, Law BF, Mild RM, Hofacre CL, Singer RS 2013 Enumeration of Salmonella and Campylobacter spp. in environmental farm samples and processing plant carcass rinses from commercial broiler chicken flocks. Appl Environ Microbiol 79:4106–4114. doi:10.1128/AEM.00836-13. 23624481 PMC3697552

[16] Kullenberg C, Kasperowski D 2016 What is citizen science? A scientometric meta-analysis. PLoS One 11:e0147152. doi:10.1371/journal.pone.0147152. 26766577 PMC4713078

[17] Bonney R, Shirk JL, Phillips TB, Wiggins A, Ballard HL, Miller-Rushing AJ, Parrish JK 2014 Next steps for citizen science. Science 343:1436–1437. doi:10.1126/science.1251554. 24675940

[18] Oliver JD 2005 The viable but nonculturable state in bacteria. J Microbiol 43:93–100. 15765062

[19] Wall T 11 11 2015 Experts fail to identify cause of mystery E. coli outbreak. Environmental Health News. http://www.ehn-online.com/news/article.aspx?id=14914.

[20] DEFRA. 2013 Livestock numbers in England and the UK. Department for Environment, Food and Rural Affairs, London, United Kingdom.

[21] Ogden ID, Dallas JF, MacRae M, Rotariu O, Reay KW, Leitch M, Thomson AP, Sheppard SK, Maiden M, Forbes KJ, Strachan NJC 2009 Campylobacter excreted into the environment by animal sources: prevalence, concentration shed, and host association. Foodborne Pathog Dis 6:1161–1170. doi:10.1089/fpd.2009.0327. 19839759 PMC3985071

[22] Doyle MP, Roman DJ 1982 Sensitivity of Campylobacter jejuni to drying. J Food Prot 45:507–510. doi:10.4315/0362-028X-45.6.507. 30866225

[23] Fernández H, Vergara M, Tapia F 1985 Desiccation resistance in thermotolerant Campylobacter species. Infection 13:197. doi:10.1007/BF01642813. 4044047

[24] Oosterom J, De Wilde GJA, De Boer E, De Blaauw LH, Karman H 1983 Survival of Campylobacter jejuni during poultry processing and pig slaughtering. J Food Prot 46:702–706. doi:10.4315/0362-028X-46.8.702. 30921893

[25] Sterk A, Schijven J, de Roda Husman AM, de Nijs T 2016 Effect of climate change on runoff of Campylobacter and Cryptosporidium from land to surface water. Water Res 95:90–102. doi:10.1016/j.watres.2016.03.005. 26986498

[26] Sinton LW, Braithwaite RR, Hall CH, Mackenzie ML 2007 Survival of indicator and pathogenic bacteria in bovine feces on pasture. Appl Environ Microbiol 73:7917–7925. doi:10.1128/AEM.01620-07. 17951435 PMC2168137

[27] Kelana LC, Griffiths MW 2003 Use of an autobioluminescent Campylobacter jejuni to monitor cell survival as a function of temperature, pH, and sodium chloride. J Food Prot 66:2032–2037. doi:10.4315/0362-028X-66.11.2032. 14627279

[28] Blaser MJ, Hardesty HL, Powers B, Wang WLL 1980 Survival of Campylobacter fetus subsp. jejuni in biological milieus. J Clin Microbiol 11:309–313. 6892819 10.1128/jcm.11.4.309-313.1980 PMC273394

[29] Sen A, Harwood AR, Bateman IJ, Munday P, Crowe A, Brander L, Raychaudhuri J, Lovett AA, Foden J, Provins A 2014 Economic assessment of the recreational value of ecosystems: methodological development and national and local application. Environ Resource Econ 57:233–249. doi:10.1007/s10640-013-9666-7.

[30] Ordnance Survey. 2013 1:25 000 Raster tiles. EDINA Digimap Service, Edinburgh, United Kingdom.

[31] Nicholson FA, Groves SJ, Chambers BJ 2005 Pathogen survival during livestock manure storage and following land application. Bioresour Technol 96:135–143. doi:10.1016/j.biortech.2004.02.030. 15381209

[32] Walsh PS, Metzger DA, Higuchi R 1991 Chelex 100 as a medium for simple extraction of DNA for PCR-based typing from forensic material. Biotechniques 10:506–513. 1867860

[33] Katzav M, Isohanni P, Lund M, Hakkinen M, Lyhs U 2008 PCR assay for the detection of Campylobacter in marinated and non-marinated poultry products. Food Microbiol 25:908–914. doi:10.1016/j.fm.2008.05.010. 18721681

[34] Klena JD, Parker CT, Knibb K, Ibbitt JC, Devane PML, Horn ST, Miller WG, Konkel ME 2004 Differentiation of Campylobacter coli, Campylobacter jejuni, Campylobacter lari, and Campylobacter upsaliensis by a multiplex PCR developed from the nucleotide sequence of the lipid A gene lpxA. J Clin Microbiol 42:5549–5557. doi:10.1128/JCM.42.12.5549-5557.2004. 15583280 PMC535264

[35] Linton D, Owen RJ, Stanley J 1996 Rapid identification by PCR of the genus Campylobacter and of five Campylobacter species enteropathogenic for man and animals. Res Microbiol 147:707–718. doi:10.1016/S0923-2508(97)85118-2. 9296105

[36] Williams NJ, Jones TR, Leatherbarrow HJ, Birtles RJ, Lahuerta-Marin A, Bennett M, Winstanley C 2010 Isolation of a novel Campylobacter jejuni clone associated with the bank vole, Myodes glareolus. Appl Environ Microbiol 76:7318–7321. doi:10.1128/AEM.00511-10. 20851991 PMC2976251

[37] Kärenlampi RI, Tolvanen TP, Hänninen ML 2004 Phylogenetic analysis and PCR-restriction fragment length polymorphism identification of Campylobacter species based on partial groEL gene sequences. J Clin Microbiol 42:5731–5738. doi:10.1128/JCM.42.12.5731-5738.2004. 15583306 PMC535295

[38] Joshi NA, Fass JN 2011 Sickle: a sliding-window, adaptive, quality-based trimming tool for FastQ files (version 1.33). https://github.com/najoshi/sickle.

[39] Martin M 2011 Cutadapt removes adapter sequences from high-throughput sequencing reads. EMBnet. journal 17:10–12. doi:10.14806/ej.17.1.200.

[40] Nurk S, Bankevich A, Antipov D, Gurevich A, Korobeynikov A, Lapidus A, Prjibelsky A, Pyshkin A, Sirotkin A, Sirotkin Y, Stepanauskas R, McLean J, Lasken R, Clingenpeel SR, Woyke T, Tesler G, Alekseyev MA, Pevzner PA 2013 Assembling genomes and mini-metagenomes from highly chimeric reads, p 158–170. *In* Deng M, Jiang R, Sun F, Zhang X (ed), Research in computational molecular biology: 17th Annual International Conference, RECOMB 2013, Beijing, China, April 7–10, 2013: proceedings. Springer, Berlin, Germany. doi:10.1007/978-3-642-37195-0_13.

[41] Langmead B, Salzberg S 2012 Fast gapped-read alignment with Bowtie 2. Nat Methods 9:357–359. doi:10.1038/nmeth.1923. 22388286 PMC3322381

[42] Pritchard JK, Stephens M, Donnelly P 2000 Inference of population structure using multilocus genotype data. Genetics 155:945–959. 10835412 10.1093/genetics/155.2.945 PMC1461096

[43] Strachan NJC, Gormley FJ, Rotariu O, Ogden ID, Miller G, Dunn GM, Sheppard SK, Dallas JF, Reid TMS, Howie H, Maiden MCJ, Forbes KJ 2009 Attribution of Campylobacter infections in northeast Scotland to specific sources by use of multilocus sequence typing. J Infect Dis 199:1205–1208. doi:10.1086/597417. 19265482 PMC3985119

[44] Met Office. 2012 Met Office Integrated Data Archive System (MIDAS) Land and Marine Surface Stations Data (1853–current). NCAS British Atmospheric Data Centre. http://catalogue.ceda.ac.uk/uuid/220a65615218d5c9cc9e4785a3234bd0.

[45] Centre for Ecology and Hydrology. 2015 National River Flow Archive, Natural Environment Research Council. http://nrfa.ceh.ac.uk/.

