# Supplementary material for: Novel Sampling Method for Assessing Human-Pathogen Interactions in the Natural Environment Using Boot Socks and Citizen Scientists, with Application to Campylobacter Seasonality
Source: Appl Environ Microbiol. 2017 Jun 30;83(14):e00162-17. doi: 10.1128/AEM.00162-17 (PMC5494624; doi:10.1128/AEM.00162-17)
Supplement: Supplemental material [file supp_83_14_e00162-17__index.html]

Novel Sampling Method for Assessing Human-Pathogen Interactions in the Natural Environment Using Boot Socks and Citizen Scientists, with Application to Campylobacter Seasonality — Supplemental material 

# Novel Sampling Method for Assessing Human-Pathogen Interactions in the Natural Environment Using Boot Socks and Citizen Scientists, with Application to Campylobacter Seasonality

## Supplemental material

- Supplemental file 1 -

  Histogram of the deviance residuals from the mixed-effect model (Fig. S1); scatter plot of the deviance residuals from the mixed-effect model against the linear predictors of the fixed-effect portion of the mixed-effect model (Fig. S2).

  PDF, 43K
