# Supplementary material for: Novel Sampling Method for Assessing Human-Pathogen Interactions in the Natural Environment Using Boot Socks and Citizen Scientists, with Application to Campylobacter Seasonality
Source: Appl Environ Microbiol. 2017 Jun 30;83(14):e00162-17. doi: 10.1128/AEM.00162-17 (PMC5494624; doi:10.1128/AEM.00162-17)

Supplementary material

Figure S1: Histogram of the deviance residuals from the mixed effects model.

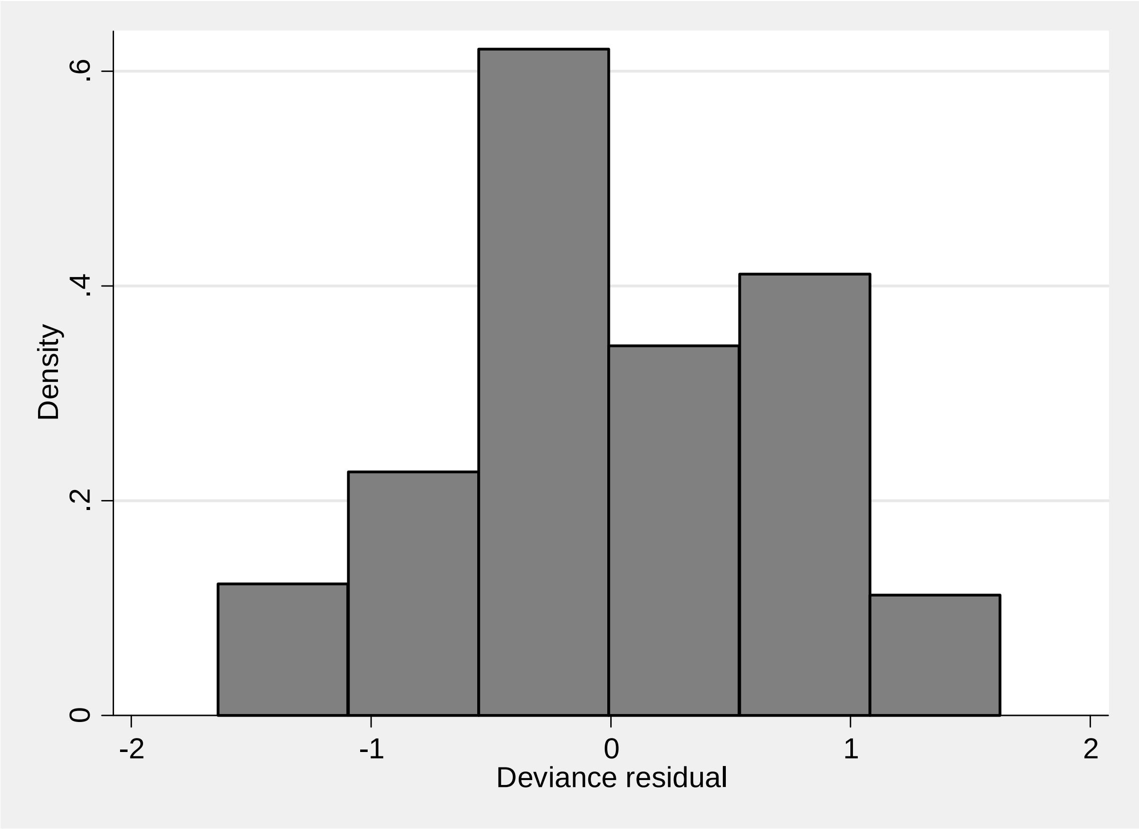

Figure S2: Scatter plot of the deviance residuals from the mixed effects model against the linear predictors of the fixed effects portion of the mixed effects model

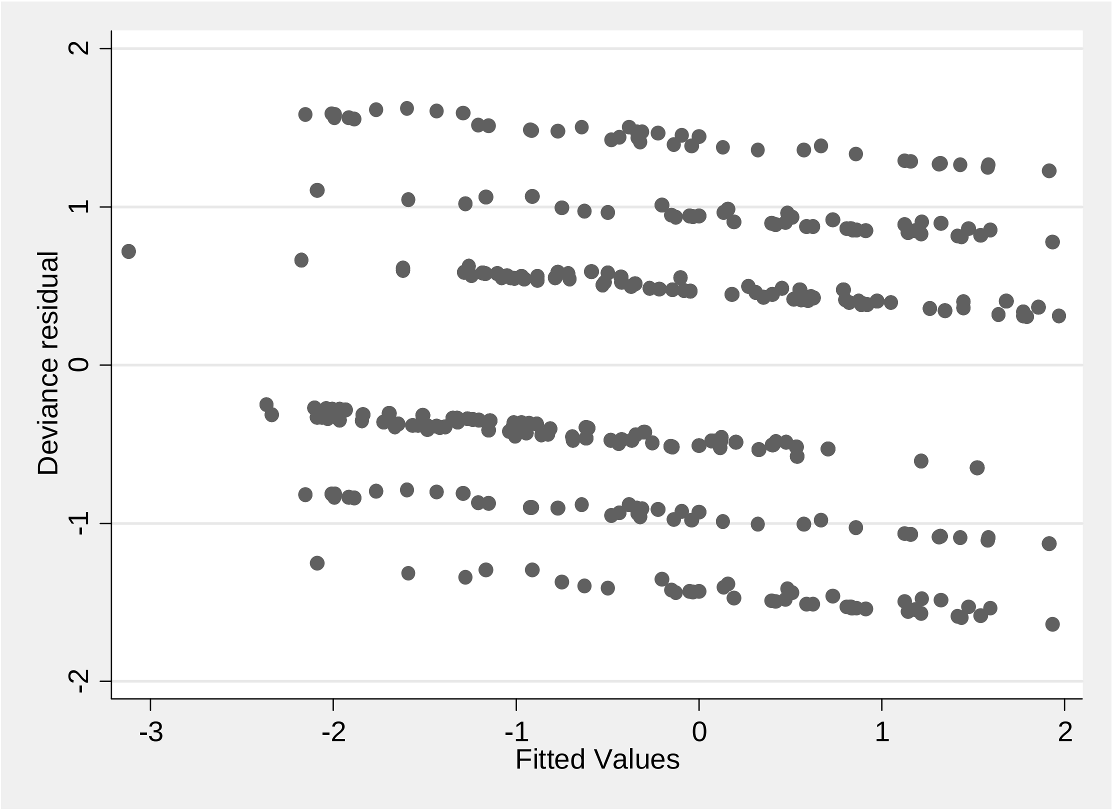

Supplement: Supplemental material [file AEM.00162-17_zam014177929s1.pdf]
